# High-yield Production of Amyloid-β Peptide Enabled by a Customized Spider Silk Domain

**DOI:** 10.1038/s41598-019-57143-x

**Published:** 2020-01-14

**Authors:** Axel Abelein, Gefei Chen, Kristīne Kitoka, Rihards Aleksis, Filips Oleskovs, Médoune Sarr, Michael Landreh, Jens Pahnke, Kerstin Nordling, Nina Kronqvist, Kristaps Jaudzems, Anna Rising, Jan Johansson, Henrik Biverstål

**Affiliations:** 1 Department of Neurobiology, Care Sciences and Society, Center for Alzheimer Research, Division of Neurogeriatrics, Karolinska Institutet, 141 52 Huddinge, Sweden; 2 Department of Physical Organic Chemistry, Latvian Institute of Organic Synthesis, Riga, LV-1006 Latvia; 3 Department of Molecular Tumor and Cell Biology, Karolinska Institutet, 171 65 Solna, Sweden; 4 Department of Pathology, University of Oslo/Oslo University Hospital, N-0424 Oslo, Norway; 5 LIED, University of Lübeck, D-23538 Lübeck, Germany; 6 Department of Pharmacology, Medical Faculty, University of Latvia, Riga, LV-1004 Latvia; 7 Department of Anatomy, Physiology and Biochemistry, Swedish University of Agricultural Sciences, 750 07 Uppsala, Sweden

**Keywords:** Biochemistry, Biophysics, Biosynthesis, Peptides, Proteins, Neurodegeneration, Neurodegenerative diseases

## Abstract

During storage in the silk gland, the N-terminal domain (NT) of spider silk proteins (spidroins) keeps the aggregation-prone repetitive region in solution at extreme concentrations. We observe that NTs from different spidroins have co-evolved with their respective repeat region, and now use an NT that is distantly related to previously used NTs, for efficient recombinant production of the amyloid-β peptide (Aβ) implicated in Alzheimer’s disease. A designed variant of NT from *Nephila clavipes* flagelliform spidroin, which in nature allows production and storage of β-hairpin repeat segments, gives exceptionally high yields of different human Aβ variants as a solubility tag. This tool enables efficient production of target peptides also in minimal medium and gives up to 10 times more isotope-labeled monomeric Aβ peptides per liter bacterial culture than previously reported.

## Introduction

Orb-weaving spiders manufacture up to seven different silks, *e.g*. dragline silk derived from major ampullate silk proteins (spidroins, MaSp) and flagelliform silk derived from flagelliform spidroins (FlSp). The various spidroins share a common architecture - a large core repetitive region capped by globular N- and C-terminal domains (NT and CT)^1^. The divergent and large aggregation-prone repetitive regions of the spidroins determine the mechanical properties of the respective spider silks, while the terminal domains regulate silk fiber formation^2,3^. Despite their high aggregation propensity the spidroins can be stored at extremely high concentrations (30–50% w/v) in the spider silk gland, solubilized by the NT domain^1,4^.

The NT dimerizes upon a drop in pH, which is crucial for silk fiber formation^1,5^. To ensure solubility also at low pH and widen the applicability of NT as a solubility enhancing fusion partner, a charged-reversed mutant has been designed (referred to as NT*_MaSp_)^6^. The previously reported NT*_MaSp_ tag is derived from the NT domain of *Euprosthenops australis* MaSp1 and folds as a five-helix bundle^6,7^. NT*_MaSp_ is a pH insensitive constitutive monomer, highly stable and extremely soluble, and has been successfully applied for efficient production and purification of, among others, lung surfactant protein analogs, cholecystokinin-58, human antimicrobial cathelicidin and a designed β-sheet protein^6,8^.

Aggregation-prone proteins and peptides are associated with several neurodegenerative disorders, *e.g*. Alzheimer’s disease (AD), the most prevalent form of dementia^9,10^. These proteins/peptides often exhibit high β-sheet propensity, which make them prone to aggregate and form insoluble amyloid fibrils^11^. These intrinsic properties of amyloid-forming proteins make high-yield biochemical production challenging, yet the availability of pure protein samples is crucial for studying protein self-assembly and its associated neurotoxicity *in vitro* and *in vivo*. This is probably one important reason behind the fact that, despite immense efforts, the exact mechanisms of Aβ self-assembly are still unknown^9–11^. Recent advances have however revealed new insights into the nucleation mechanism of Aβ *in vitro*^12,13^, structural details of the fibril morphology^14^ and biological mechanisms implicated in the AD etiopathology^15,16^. These experiments typically require access to very pure and homogeneous Aβ peptides as small impurities or preformed seeds have a great impact on the aggregation behavior^17^. In particular for structural studies of amyloid fibrils, but also for certain *in vivo* experiments, the availability of large quantities of isotope-labeled Aβ is essential.

Studies of Aβ aggregation *in vitro* have often been conducted with synthetically produced peptides^18,19^. Synthetic preparations have several drawbacks including batch-to-batch variations, intrinsic impurities and relatively high cost, especially for isotope labeling. As a consequence, several recombinant expression systems have been established. These production protocols either result in peptides with an initiating non-native methionine residue^20,21^ or are based on solubility tags that require proteolytic cleavage to obtain the native human Aβ sequence^22–24^. The main disadvantage of having methionine as the first residue is that it might affect processes such as posttranslational modifications, *e.g*. pyroGlu formation^25–27^, and metal ion binding since the metal ion-binding site is located in the N-terminus^28–30^. Here we describe a useful solubility tag for production of aggregation-prone proteins and peptides, and demonstrate that this tool enables very efficient production of native and isotope-labeled Aβ peptides.

## Results and Discussion

### Evolutionary relationships of NT and repetitive regions of different spidroins

**Figure 1:**
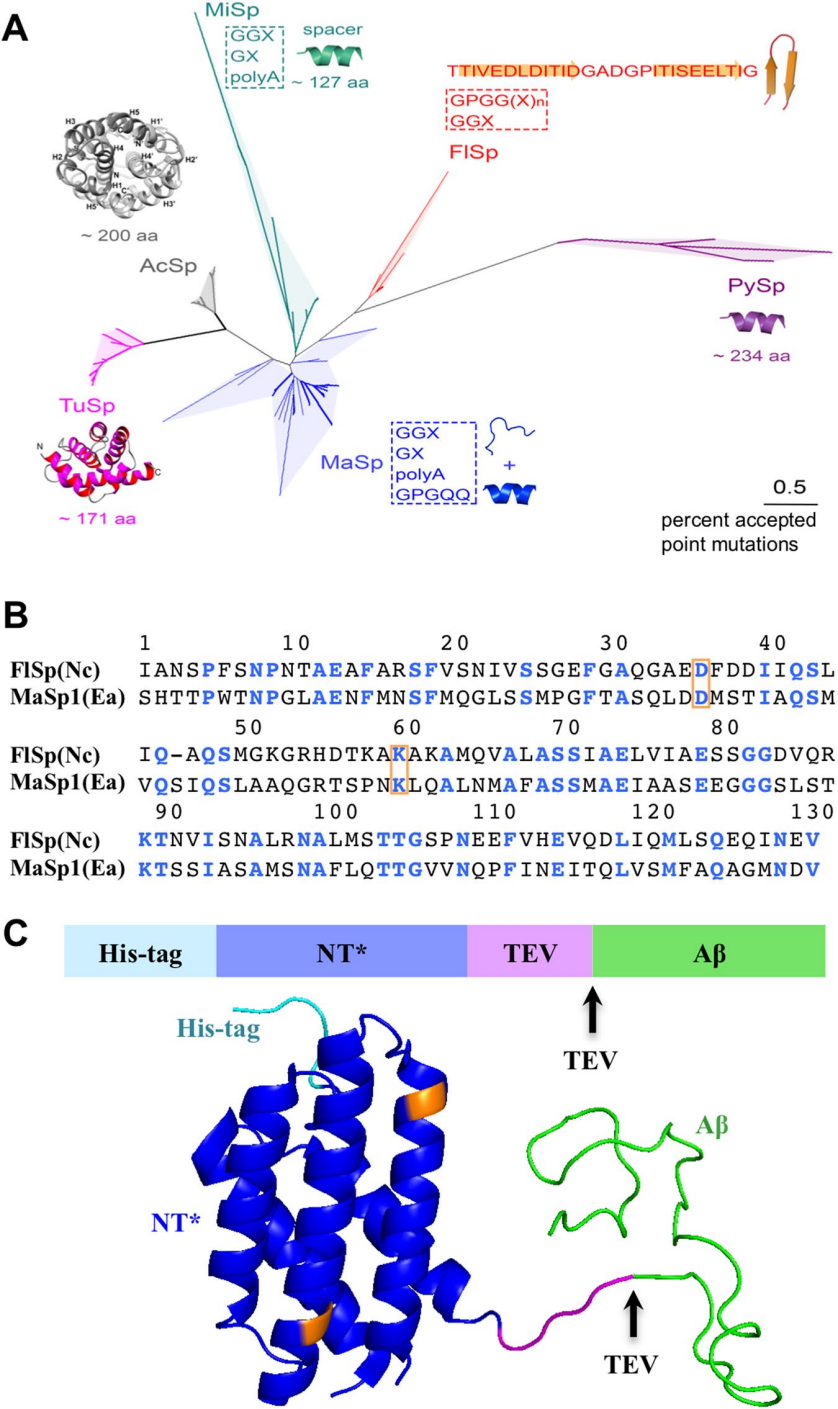
(**A**) Evolutionary relationships of the NTs of different spidroins. The analysis involved 67 NT amino acid sequences (Supplementary Fig. 1), revealing that spidroins from different spider species cluster according to the silk type in the phylogenetic tree. The typical repetitive regions of the respective spidroins and their known structures or main secondary structure propensities are displayed. (**B**) Sequence alignment of NT_FlSp_ and NT_MaSp_ where strictly conserved residues are colored in blue. The residues marked in orange display the mutated sites in NT*. (**C**) Schematic representation and structure of the NT*-Aβ fusion protein where the arrows indicate the TEV protease cleavage site. The mutated D and K residues are marked by yellow colour in the NT structure (pdb 4FBS).

A phylogenetic tree based on sequence alignment of 67 NTs found in GenBank (Supplementary Fig. S1) reveal evolutionary relationships between NT and their respective repetitive regions (Fig. 1A). The NT domains cluster according to silk type, as previously reported^7^. Hence, the NTs of different spidroin types, which are defined by the nature of their respective repetitive regions, have been conserved through evolution of different spider species. Structural characteristics of the repetitive regions appear to co-vary with the evolution of NTs, *e.g.* for the tubuliform (TuSp) and aciniform (AcSp) NTs, which are evolutionarily close, the corresponding repetitive regions stand out by forming globular folded domains^31,32^ (Fig. 1A). NT from FlSp is linked to a unique repetitive region that contains several embedded spacers (each 27 residues), which are predicted to form β-hairpins^33^ (Fig. 1A). NT_FlSp_ exhibits distant evolutionary relationship (<35% sequence identity) to the previously reported NT_MaSp_^6^ (Fig. 1B) and MaSps contain repetitive regions with predicted α-helical and random coil structures^34–38^. We speculate that different NTs may have evolved to facilitate optimal solubility of their respective repeat region in the silk gland during storage conditions, where pH is neutral and NT monomeric^4,5^. Irrespective of any potential evolutionary co-variation between NTs and the repetitive regions, we aimed to explore whether NT_FlSp_ could work in protein expression in an equivalent way to the previously investigated and distantly related NT_MaSp_^6^.

### Design of the novel solubility tag NT*_FlSp_

To prevent dimerization of NT_FlSp_ at low pH we introduced the D40K and K65D mutations^6^ in NT_FlSp_ from *Nephila clavipes (Nc)* (Fig. 1B) (numbering as described previously^6^, wherefore the mutations correspond to positions 36 and 60 in Fig. 1B). NT*_FlSp_ has a larger number of charged residues (25 *vs*. 11) and stronger net charge (−7 *vs*. −5) compared to NT*_MaSp_, which potentially enhance its solubility properties. In contrast to NT*_MaSp_, NT*_FlSp_ has no tryptophan, whose absorbance at 280 nm would cover the intrinsic low absorbance at 280 nm of the target peptide Aβ. Thus, for size exclusion chromatography (SEC), where detection relies on the protein absorbance at 280 nm, NT*_FlSp_ enables clearly separated intensity peaks.

### Efficient expression and purification of Aβ monomers using NT*_FlSp_

**Figure 2:**
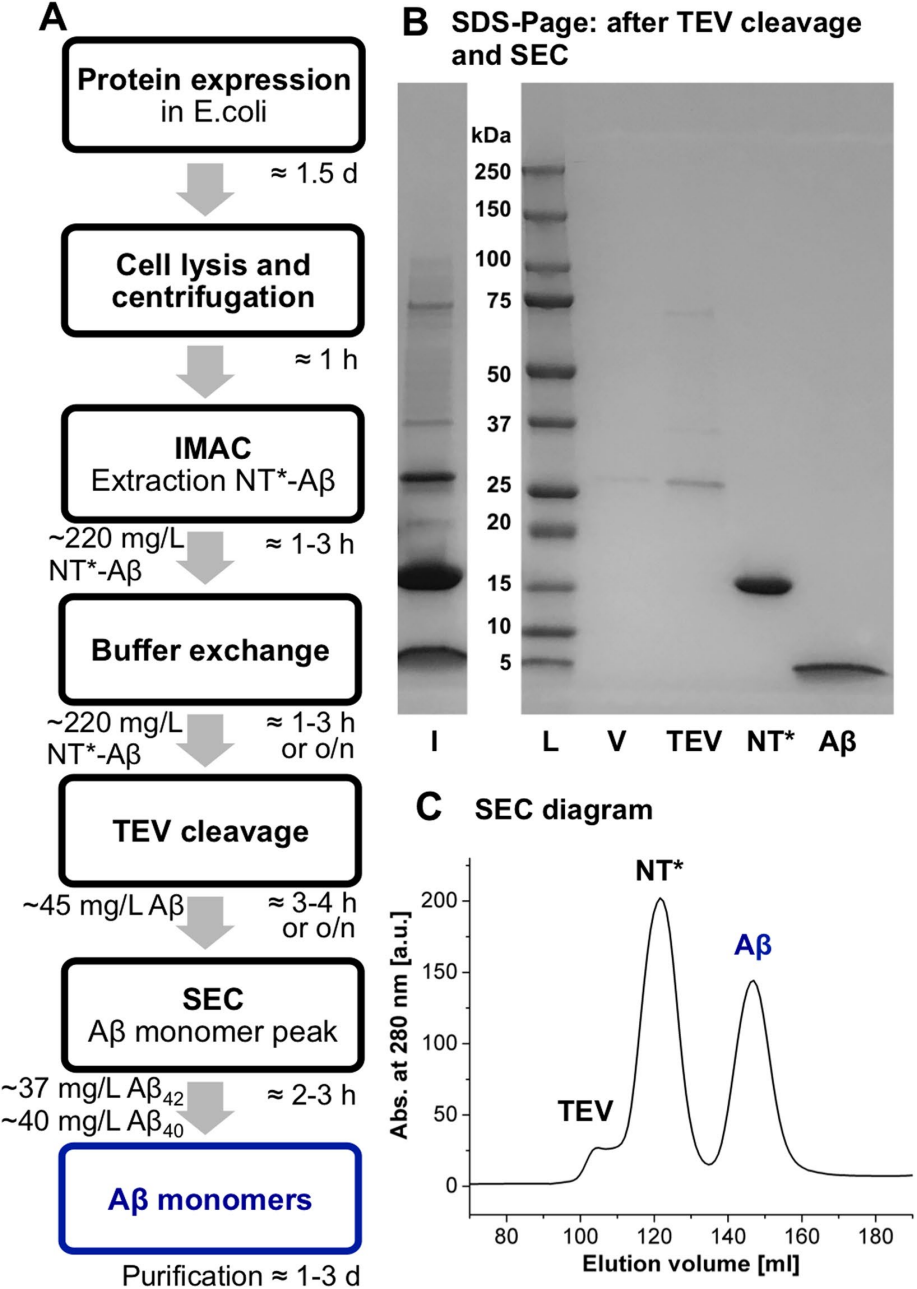
(**A**) Schematic expression and purification protocol, including typical times for performance. Yields of NT*-Aβ_42/40_ are derived from 1 L expression cultures and extrapolated from purification from 100 and 500 mL, resulting in very similar values. (**B**) SDS-PAGE gel, with protein ladder (L), void (V), before (I) and after SEC yielding pure Aβ. An uncropped full-length gel is presented in Supplementary Fig. S2. (**C**) SEC diagram showing separation of TEV, NT* and monomeric Aβ.

We designed the fusion protein NT*_FlSp_-Aβ by fusing the genetic codes of the solubility tag NT*_FlSp_ and Aβ with a TEV recognition site in-between (Fig. 1C). An overview of the expression and purification scheme is given in Fig. 2. The fusion protein was expressed in BL21(DE3) *E.coli* cells grown in rich or minimal medium, dissolved in 8 M urea after cell lysis and purified using immobilized metal ion affinity chromatography (IMAC). Urea was added as denaturant to increase binding to IMAC column. For optimal cleavage of the fusion protein by TEV, a buffer exchange was conducted, either by overnight dialysis or by column chromatography. TEV cleavage can alternatively be conducted during buffer dialysis to speed-up the purification, yet a short dialysis step to decrease the urea concentration below 2 M is recommended before the addition of TEV protease. Finally, the solution was applied to SEC with a Superdex 30 column, whereby monomeric Aβ monomers were isolated.

The expression and purification protocol presented here results in highly pure Aβ_40_ and Aβ_42_ monomers within 2.5–4.5 days, depending on buffer exchange and cleavage method. The yields in rich and minimal medium are listed in Table 1. The example shown in Fig. 2 represents purifications from 100 and 500 mL culture medium, yielding very similar amounts of 37 ± 7 mg of pure Aβ_42_ monomers if extrapolated to one-liter culture. Notably, the present scheme gives by far the highest yields, both in rich and minimal medium, compared to other reported protocols (Table 2).

**Table 1:** Average yields of fusion proteins and monomeric Aβ peptides in rich (LB) and minimal (M9) medium in mg per liter culture.

Protein/peptide	Rich medium [mg/L]	M9 medium [mg/L]
NT*_FlSp_-Aβ_40_	216 ± 29	74 ± 22
NT*_FlSp_-Aβ_42_	223 ± 42	88 ± 10
Monomeric Aβ_40_	40 ± 5	13 ± 4
Monomeric Aβ_42_	37 ± 7	14 ± 2

Errors were estimated as standard deviations from 5 replicates by western blot analysis (see Methods).

**Table 2:** Yields of Aβ_40_ and Aβ_42_ variants reported in literature and herein.

Aβ variant	Fusion partner/expression method	Purified Aβ peptide yield in mg/L in rich medium	Purified Aβ peptide yield in mg/L in minimal medium	Reference
Aβ(1–40)	NT*_FlSp_	40 ± 5	13 ± 4	here
	(NANP)_19_	22	—	^22^
	IFABP	4	—	^24^
	GST	7	1.5	^23^
Aβ(M1–40)	Directly from inclusion bodies	10–20	—	^21^
	Directly from inclusion bodies	—	10–15	^51^
	Co-expression with Z_Aβ3_	—	4	^20^
Aβ(1–42)	NT*_FlSp_	37 ± 7	14 ± 2	here
	(NANP)_19_	19	—	^22^
	IFABP	3	—	^24^
	IFABP	—	6	^52^
	GST	15	—	^53^
	Ub	4	—	^54^
Aβ(M1–42)	Directly from inclusion bodies	8	—	^21^
	Co-expression with Z_Aβ3_	—	3	^20^

**Figure 3:**
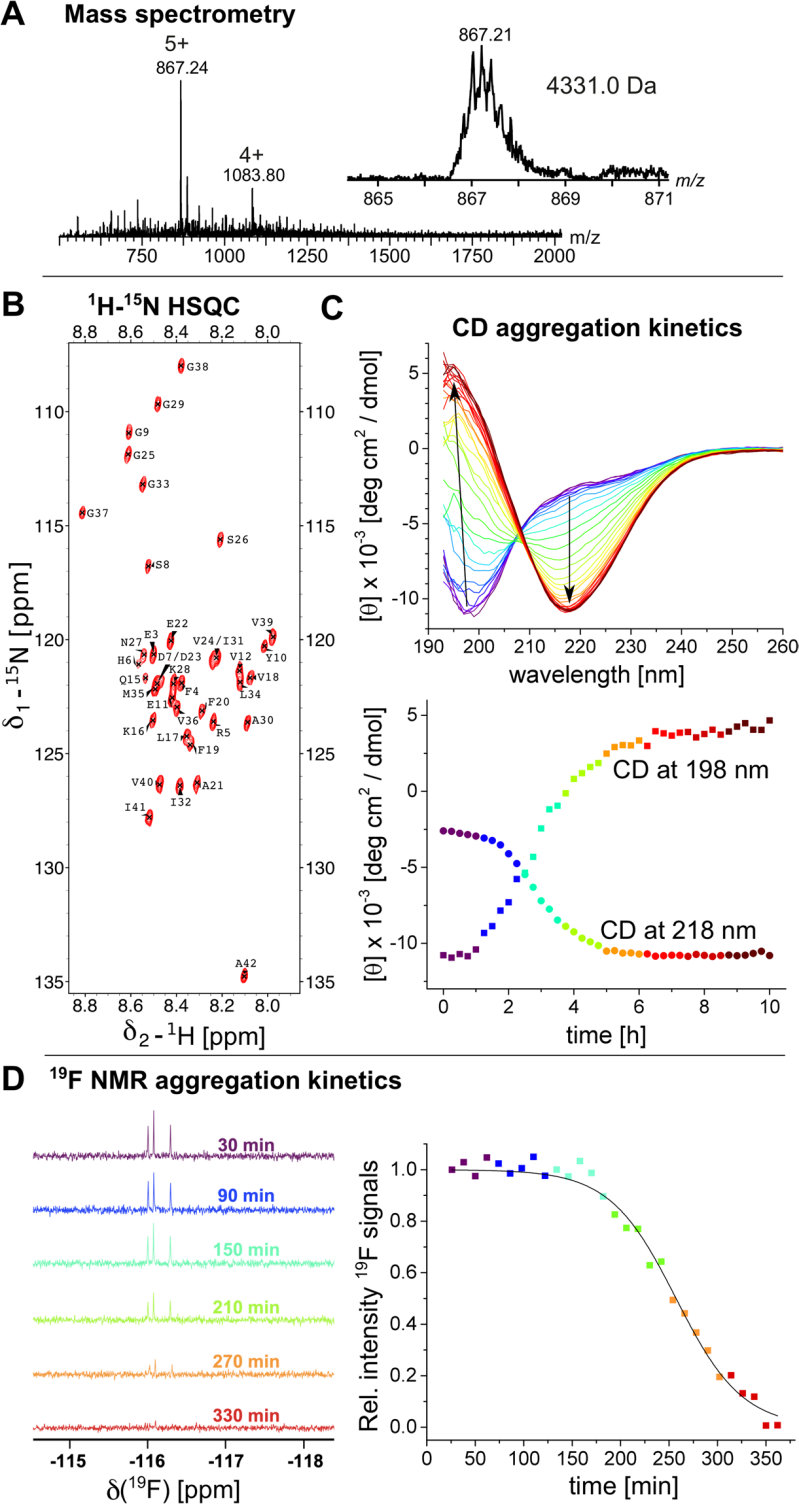
(**A**) Mass spectrum of Aβ_40_ showing a pure peptide with an average mass of 4331 Da. The inset shows a zoom of the 5 + charged ion. (**B**) ^1^H-^15^N-HSQC spectrum of 15 μM Aβ_42_ recorded at 5 °C, revealing monomeric peptide. (**C**) Aggregation kinetics of 10 μM Aβ_42_ at 37 °C under continuous stirring recorded by CD spectroscopy. The spectra exhibit a structural transition from a predominantly unstructured state to a β-structure. The lower panel shows the time dependence of the CD extremes at 198 nm (squares) and 218 nm (circles), with the same color code as used for the CD spectra. (**D**) Aggregation kinetics of 50 μM 4FF-Aβ_42_ at 25 °C monitored by ^19^F-NMR spectra of the signals around −116 ppm, exhibiting attenuation of 4FF-signals. The color code represents the same time points in both panels.

The purified peptides were investigated using mass spectrometry, confirming the expected masses for Aβ, here shown for Aβ_40_ (Fig. 3A). Using ^13^C-^15^N-double-labeled Aβ_40_ and Aβ_42_ we performed nuclear magnetic resonance (NMR) experiments to confirm the purity and structural state of the purified peptides. We recorded ^1^H-^15^N-HSQC experiments (Fig. 3B and Supplementary Fig. S3) where the chemical shifts of the cross-peaks coincide with previous assignments reported in the literature, revealing a monomeric, predominantly unstructured conformation of the purified Aβ peptides^39,40^. To analyze the secondary structure of monomeric Aβ_42_ we applied circular dichroism (CD) spectroscopy. The initial CD spectra (Fig. 3C) indicated a predominantly unstructured conformation as previously reported^20,21,41,42^. Taken together, these experiments confirm that our method results in monomeric Aβ_40_ and Aβ_42_ peptides.

### Production of 4-fluoro-Phe-labeled Aβ peptides

The present approach also opens new opportunities for NMR studies that require more complex isotope labeling approaches associated with reduced protein yields. For example, we have used the NT*_FlSp_ tag to produce monomeric Aβ_42_ incorporating 4-fluoro-Phe (4FF-Aβ_42_) in milligram yields. The expression was performed similarly as described above, but glyphosate and DL-tyrosine was supplemented to bacterial cultures at an OD_600nm_ value of 0.6. Further, DL-4-fluorophenylalanine was added when the OD_600nm_ value reached 0.8 and cell expression was induced. The ^1^H-^15^N-HSQC spectrum of ^15^N-labeled 4FF-Aβ_42_ revealed again a monomeric peptide (Supplementary Fig. S3).

### Aggregation kinetics of native and isotope-labeled Aβ_42_

To ensure that the isolated peptides behave as expected, we investigated the aggregation kinetics starting from monomeric Aβ peptides. Recording CD signals under continuous stirring at 37 °C of 10 μM Aβ_42_, a structural conversion from an unstructured to a β-structured conformation was observed (Fig. 3C), where the isodichroic point at 208 nm indicates a two-state transition. Furthermore, we used 50 μM 4FF-Aβ_42_ for real-time aggregation ^19^F-NMR studies at 25 °C, revealing a decrease of 4FF-signals over time (Fig. 3D). The signal loss can be fitted to a sigmoidal decline, with an aggregation half time of 258 ± 5 min under the conditions used.

**Figure 4:**
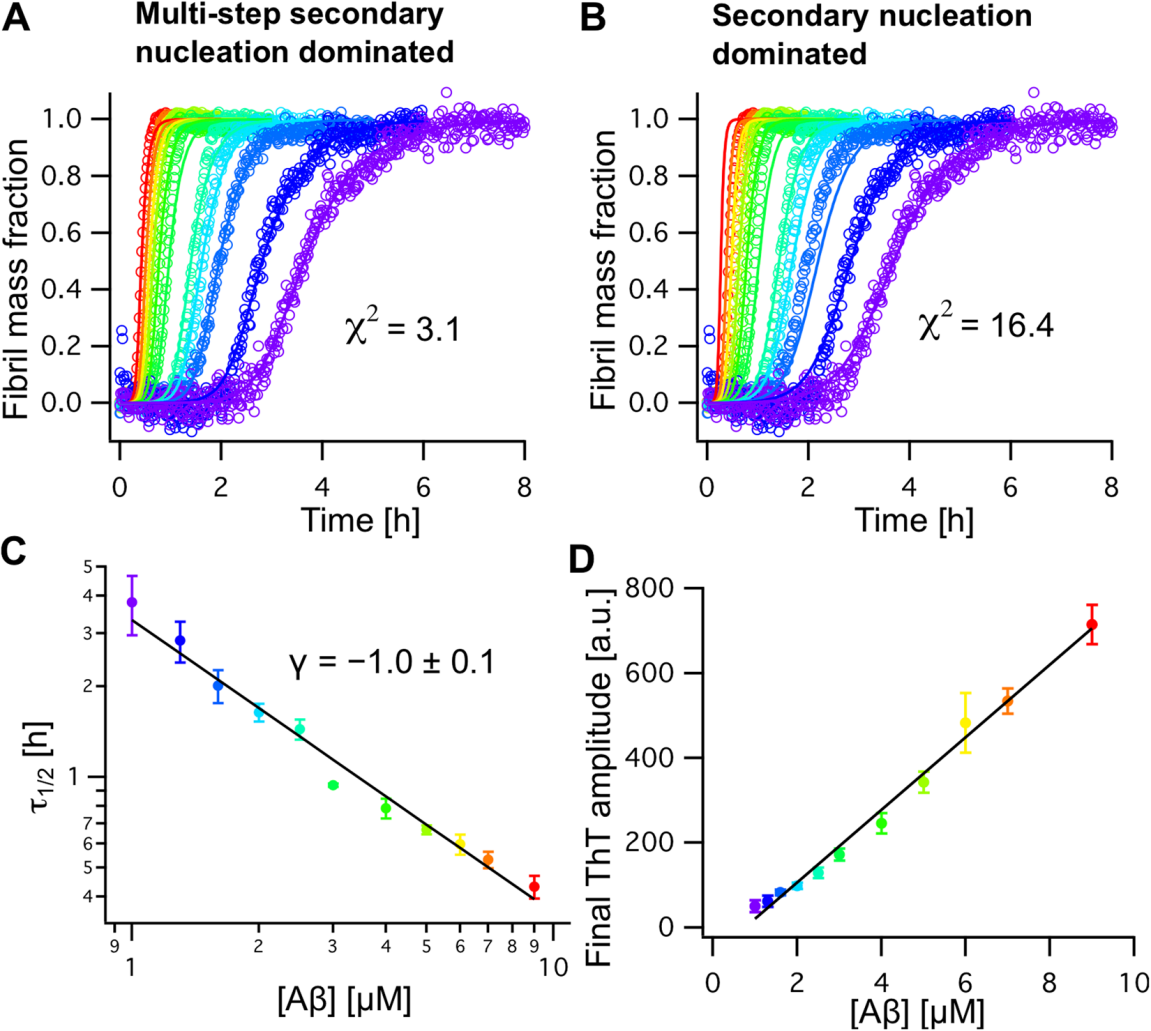
(**A,B**) Aggregation kinetics of Aβ_42_ at different concentrations from 1.0 (violet) to 9.0 μM (red) recorded by ThT fluorescence experiments fitted with a multi-step (**A**) and simple secondary nucleation model (**B**). The kinetic traces fit best to the multi-step secondary nucleation model, reflected by a lower χ^2^ value. (**C**) Aggregation half times, 𝜏_1/2_, plotted against the initial peptide concentration, [Aβ], exhibit a *γ* coefficient of *γ* = −1.0 ± 0.1. (**D**) The final ThT intensity of the normalized aggregation traces in (**A**) exhibits a linear relation to [Aβ].

Alternatively, Aβ aggregation kinetics can be monitored using the fluorescence dye thioflavin T (ThT), for a detailed elucidation of the nucleation mechanism. Here, we conducted ThT experiments on Aβ_42_ in 20 mM sodium phosphate buffer, pH 8.0, at 37 °C under quiescent conditions at different initial Aβ_42_ monomer concentrations, [Aβ] (Fig. 4). The final fluorescence intensity exhibits a linear dependence on the initial monomer concentration (Fig. 4D), suggesting that the total amount of initially monomeric peptides forms ThT-active fibril material, as previously shown for Aβ^12,13,42^. The aggregation half times of Aβ_42_ used here exhibit a simple relation *τ*_1/2_ ∝ [Aβ]^*γ*^, with *γ* = −1.0 ± 0.1, corresponding to the slope in a double logarithmic plot (Fig. 4C). This value is in the same range as found for Aβ_M42_ with an initial methionine, where *γ* = −1.3 was reported^12^. For *γ* = −1.0 a multi-step secondary nucleation model describes better the observed aggregation traces compared to a single-step secondary nucleation model (Fig. 4A,B). The multi-step model additionally includes saturation of secondary nucleation and was previously shown to be applicable for the shorter Aβ_40_ and Aβ_M40_ variants^13,29,42^ and for Aβ_M42_ at pH 7.4^43^, which all exhibit higher *γ*-values, but also describes well the kinetics of Aβ_M42_ at pH 8.0^44^. Hence, these results confirm that the native and isotope-labeled peptides obtained herein are highly pure and in a monomeric state, which is essential for accurate and reproducible aggregation kinetics experiments.

## Conclusions

Taken together, we have developed a biomimetic tool that provides facile, fast and inexpensive production of pure and monomeric Aβ_40_ and Aβ_42_ peptides. The high yield obtained also in minimal medium enables efficient generation of isotope-labeled Aβ peptides. Peptides produced by our protocol recapitulate the behavior of Aβ peptides obtained by other means, which indicate the applicability of using NT*_FlSp_ for generating functional Aβ peptides. The NT*_FlSp_-tag holds great potential, also when compared to NT*_MaSp_^6^, for efficient production of medically relevant aggregation-prone peptides and proteins. This is important since the majority of new pharmaceuticals are biologics and facile protocols for efficient production of proteins that are difficult to produce are needed.

## Methods

### Expression and purification protocol

The synthetic gene coding for NT FlSp from *Nephila clavipes* with the D40K and K65D mutations (NT*_FlSp_) was ordered from GenScript (GenScript Biotech, Netherlands). The NT*_FlSp_ gene was ligated into pT7 plasmid containing TEV recognintion site (TRS)-Aβ40/Aβ_42_ as described previously^6^. The plasmids were transformed into chemically competent *E. coli* BL21 (DE3) cells and expressed as described previously^45^. In short, 1 mL overnight culture was inoculated to 100 mL LB medium (1/100) or 100 mL M9 overnight culture was inoculated to 1 L M9 minimal medium (10/100) with 70 mg/l kanamycin. Cells were grown at 30 °C at 120 RPM to OD_600nm_ around 0.8–0.9, where the temperature was lowered to 20 °C, and 0.1 mM Isopropyl β-D-1-thiogalactopyranoside (IPTG) was added and the cells were incubated overnight. To isolate the cells from media, the bacterial culture was centrifuged at 5,000 × g for 20 minutes at 4 °C and the cell pellets resuspended in 40 mL 20 mM Tris-HCl pH 8.0, split in to two 50 mL falcon tubes and stored at −20 °C. The frozen cells were thawed and urea was added to a concentration of 8 M. The cells were sonicated to obtain a clear solution. The lysate was then loaded on 2 × 5 mL Ni-NTA column (GE Healthcare). Unbound proteins were washed away with 15 mM Imidazole in 20 mM Tris-HCl, pH 8 and 8 M urea. The fusion protein was eluted with 300 mM imidazole in 20 mM Tris-HCl, pH 8 and 8 M urea. The fractions containing the fusion protein were pooled and then dialyzed overnight against 20 mM Tris-HCl pH 8.0 at 4 °C. To remove the His6-NT*_FlSp_ part, the fusion protein was cleaved with TEV protease (1:20–1:30, enzyme to substrate, w/w) at 4 °C overnight in 20 mM Tris-HCl pH 8, 0.5 mM EDTA and 1 mM DTT. After TEV cleavage, the sample was dissolved in 15 mL 7 M guanidine-HCl and separated on a Superdex 30 26/600PG size exclusion column (Fig. 2C). The correct size of Aβ, NT*_FlSp_ and TEV was confirmed by SDS/PAGE in a 4–20% polyacrylamide gel, stained with Coomassie brilliant blue dye (Fig. 2B). For expression of ^15^N- and ^13^C- labelled NT* _FlSp_ -Aβ, the same procedure was used except that M9 minimal medium containing ^15^NH_4_Cl (1 g/L M9) and ^13^C-glucose (4 g/L M9) was used. The plasmid pRK793 for TEV expression was obtained from addgene (addgene.org, deposited by David S. Waugh) and was expressed as described above and purified as described previously^46^.

### Determination of yields

Both NT*_FlSp_-Aβ_40_ and NT*_FlSp_-Aβ_42_ was transformed into BL21 (DE3) *E. coli* cells and spread onto an agar plate with kanamycin. 5 starting cultures of LB and M9 were inoculated with individual colonies and incubated at 31 °C overnight. The expression was performed as described above in 100 mL LB and M9 media. 100 μL of each culture was taken before and after induction, lyophilized, dissolved in SDS loading buffer and boiled for 10 minutes at 96 °C. 1 μL of each induced sample and 1 μL uninduced sample from each condition was loaded on a 4–20% mini protean TGX gel (BioRad) and blotted on a PVDF membrane (GE healthcare). 5% w/v non-fat dry milk/PBS was used to block the membrane after blotting for 1 h, followed by incubation with 6E10 primary antibody in 5% w/v non-fat dry milk, 0.1% Tween/PBS overnight at 4 °C. The membranes were washed three times with 0.1% Tween/PBS, and ECL anti-mouse secondary antibodies in 5% w/v non-fat dry milk and 0.1% Tween/PBS were added for 1 h at room temperature. Enhanced chemiluminescence detection reagent (GE Healthcare) was added and images were acquired using an AI600 imaging system (GE healthcare). The concentration of each sample was calculated by integration of the peaks from IMAC (fusion protein) and SEC (monomeric Aβ) with an extinction coefficient ε_280_ = 2,980 M^−1^cm^−1^ for the fusion protein and 1,424 M^−1^ cm^−1^ for Aβ. Western blot intensities were analyzed by ImageJ software^47^ and average and standard deviation from 5 replicates was calculated using yields from one full purification of each condition. Values are listed in Table 1.

### Expression protocol of 4FF-Aβ42

The plasmid pT7His6NT*_FlSp_-TEV recognition site -Aβ_42_ was transformed into chemically competent *E. coli* BL21(DE3) cells. Colonies were inoculated to 10 mL LB medium with 70 mg/L kanamycin and grown at 30 °C and 200 r.p.m. to OD_600nm_ > 1.0. 0.5 mL day culture was inoculated to 25 mL M9 medium with 70 mg/l kanamycin and grown at 30 °C and 200 r.p.m. overnight. 10 mL overnight culture was inoculated to 1 L M9 medium and cells were further grown at 30 °C. Uniform labeling with 4-fluorophenylalanine was achieved by the introduction of 1 g/L glyphosate and 75 mg/L DL-tyrosine to shaking bacterial cultures at 30 °C which had reached an OD_600nm_ of 0.6. Once cells achieve an OD_600nm_ of 0.8, 30 mg/L DL-4-fluorophenylalanine was added. The incubation temperature was lowered to 20 °C and expression was induced with the addition of IPTG to 0.1 mM, the cells were incubated overnight and were harvested by 7,000 × g centrifugation at 4 °C.

### Evolutionary relationships of the NT domains of different spidroins

The evolutionary history of the NT domains from different spidroins was inferred by the Neighbor-Joining method with the Poisson correction. Evolutionary analyses were conducted in MEGA7^48^. The analysis involved 67 amino acid sequences. In the spider silk gland (liquid protein), the repetitive region of MaSp, consisting of GGX, polyA, GX and GPGQQ, is disordered and partially helical^34–38^, and MiSp and FlSp share identical motifs^33^. However, there are ∼127-aa spacer in MiSp, which adopt α-helical conformation, whereas the 27-aa spacer in FlSp is predicted to fold to β-hairpin^33^. The large repetitive domains of AcSp and TuSp adopt α-helical conformation^31,32^, and the repetitive domains of PySp is also predicted to adopt α-helical conformation by PSIPRED v3.3 (http://bioinf.cs.ucl.ac.uk/psipred/).

### Mass spectrometry

Purified Aβ_40_ was diluted 1:10 in H2O/acetonitrile/formic acid (70:30:0.2) and directly infused into a Waters LCT Time of flight mass spectrometer (MS Vision, NL) equipped with an offline nanospray source using borosilicate capillaries (Thermo Scientific). The capillary voltage was 1.5 kV and the cone voltage was 200 V. Spectra were acquired between m/z 500 and 4000 and the mass scale was calibrated with Cesium Iodide. Data were analyzed using MassLynx 4.1 (Waters).

### Nuclear magnetic resonance (NMR)

^1^H-^15^N HSQC spectra were recorded on a 500 MHz or 700 MHz Bruker Avance spectrometer equipped with cryogenic probes. The HSQC spectrum of Aβ_40_ was recorded at 500 MHz at 8 °C using 75 μM peptide concentration in 16 mM sodium phosphate buffer, pH 7.4, with 0.02% NaN_3_ and 0.2 mM EDTA. For Aβ_42_ the peptide concentration was 15 μM in 20 mM sodium phosphate buffer, pH 6.8, recorded at 5 °C and 700 MHz. The spectra were recorded using 2048 × 128 complex points and 32 scans per transient. For Aβ_42_ we recorded the HSQC at 15 μM directly after the SEC purification, ensuring the monomeric state of the peptide.

^19^F-NMR experiments were recorded using 50 μM 4FF-Aβ_42_ in 20 mM sodium phosphate buffer, pH 7.4 with 0.03% NaN_3_ and 1 mM EDTA at 25 °C and 565 MHz. ^19^F spectrum was acquired with  512 transients and 1.0 s pulse delay between each transient. Line broadening of 1.0 Hz was used to process the final spectrum. The ^1^H-^15^N HSQC spectrum of 15 μM 4FF-Aβ_42_ in 20 mM sodium phosphate buffer, pH 7.4, with 0.02% NaN_3_ and 0.2 mM EDTA, was recorded at 4 °C on a 600 MHz Bruker Avance Neo spectrometer equipped with a cryogenic probe.

### Circular dichroism (CD)

CD measurements of 10 μM Aβ_42_ in 20 mM sodium phosphate buffer, pH 8.0, at 37 °C were performed in a quartzglass Suprasil 10 × 4 mm CD cuvette (Hellma Analytics) where the optical path length was 4 mm, using a Chirascan CD spectrometer (Applied Photophysics). A resolution of 1.0 nm and a bandwidth of 1 nm were chosen for the aggregation kinetics experiments^42^. During the enire measurement the sample was continuously stirred at around 1200 r.p.m and each 3 min a new CD spectrum was recorded to follow the aggregation kinetics.

### Thioflavin T (ThT) fluorescence kinetics experiments

For ThT aggregation kinetics experiments 1 to 9 μM monomeric Aβ_42_ was used, which was obtained after SEC purification^45^. ThT fluorescence was measured as described previously^45^ using 96-well microplates, where each well contained 80 μl sample solution with 10 μM ThT.

### Analysis ThT aggregation kinetics

Aggregation traces were first analyzed using a fit to a sigmoidal function, revealing the aggregation half time, τ_1/2_^29,42,45^. Subsequently, the aggregation traces were normalized and averaged over six replicates. The averaged aggregation half times are related to the initial monomer concentration, [Aβ], by τ_1/2_ ∝ [Aβ]^γ^ where γ reflects the slope in a double-logarithmic plot (Fig. 4C). Further, we applied a nucleation model including primary and secondary nucleation in addition to fibril-end elongation^12,49,50^. In order to account for saturation of secondary nucleation an equilibrium constant (Michaelis constant) *K*_*M*_ can be introduced, referring to a multi-step secondary nucleation model^13^. The kinetic equations for the time dependence of the fibril mass fractions for the two models can be found in refs. ^12,13,29,45^. The models were applied to describe the kinetic traces using a global fit analysis^12,13^. The kinetic fitting parameter are listed in Supplementary Table S1.

## Supplementary information

Supplementary Information.
