# Supplementary material for: High-yield Production of Amyloid-β Peptide Enabled by a Customized Spider Silk Domain
Source: Sci Rep. 2020 Jan 14;10:235. doi: 10.1038/s41598-019-57143-x (PMC6959368; doi:10.1038/s41598-019-57143-x)
Supplement: Supplementary file 1 — Supplementary Information. [file 41598_2019_57143_MOESM1_ESM.pdf]

Supplementary Information

High-yield Production of Amyloid-β Peptide Enabled by a Customized Spider Silk Domain

Axel Abelein[1], Gefei Chen[1], Kristīne Kitoka[2], Rihards Aleksis[2], Filips Oleskovs[2], Médoune Sarr[1], Michael Landreh[3], Jens Pahnke[4,5,6], Kerstin Nordling[1], Nina Kronqvist[1], Kristaps Jaudzems[2], Anna Rising[1,7], Jan Johansson[1], Henrik Biverstål[1,2,*]

[1]Department of Neurobiology, Care Sciences and Society, Center for Alzheimer Research, Division of Neurogeriatrics, Karolinska Institutet, 141 52 Huddinge, Sweden

[2]Department of Physical Organic Chemistry, Latvian Institute of Organic Synthesis, Riga LV-1006, Latvia

[3]Department of Molecular Tumor and Cell Biology, Karolinska Institutet, 171 65 Solna, Sweden

[4]Department of Pathology, University of Oslo/Oslo University Hospital, N-0424 Oslo, Norway

[5]LIED, University of Lübeck, D-23538 Lübeck, Germany

[6]Department of Pharmacology, Medical Faculty, University of Latvia, Riga LV-1004, Latvia

[7]Department of Anatomy, Physiology and Biochemistry, Swedish University of Agricultural Sciences, 750 07 Uppsala, Sweden

SUPPLEMENTARY FIGURES

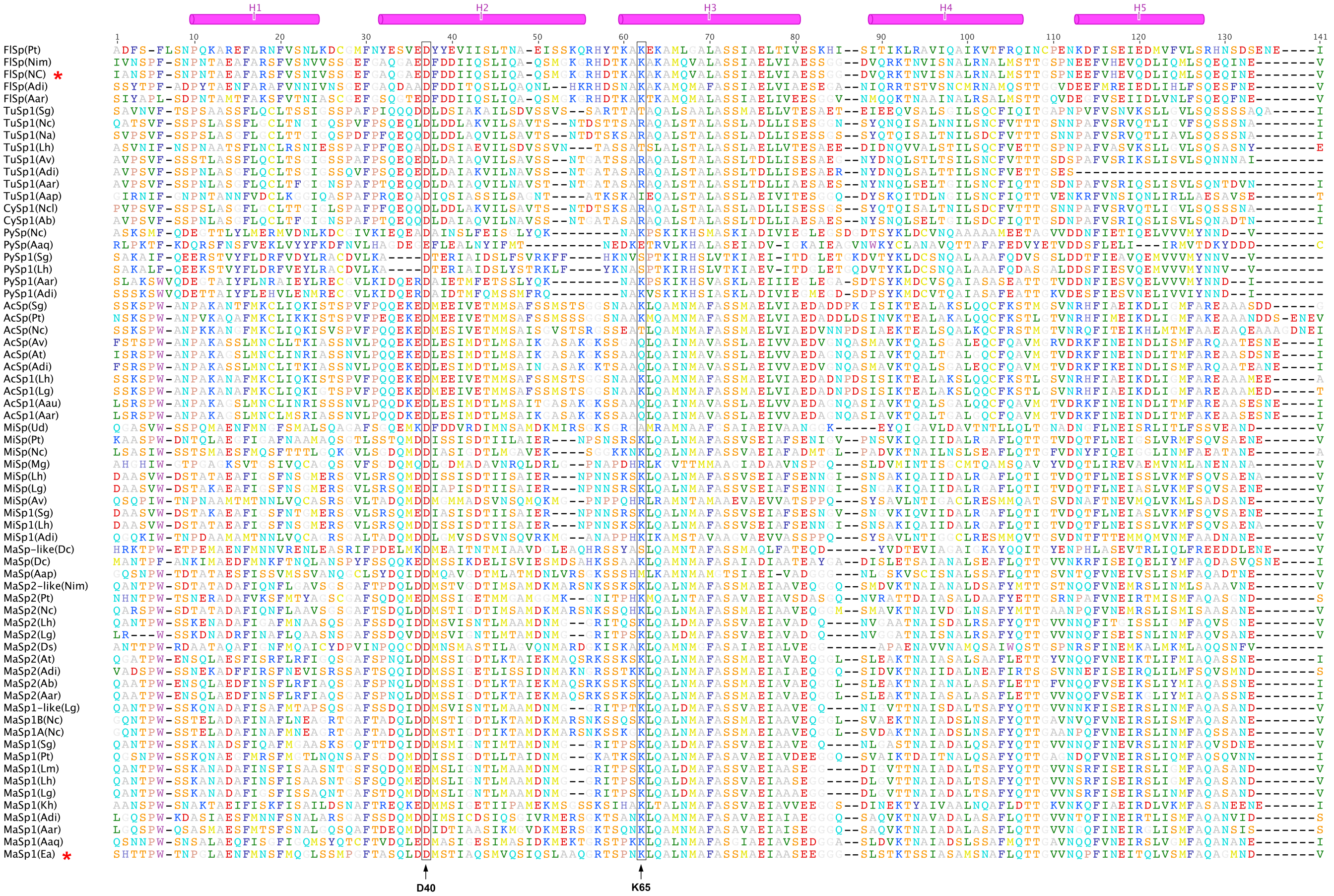

Supplementary Figure S1. Sequence alignments of non-repetitive N-terminal domains of different spidroins. The non-repetitive N-terminal sequences are aligned by Geneious Alignment and amino acids are shown in different colors. Locations of α-helices shown in pink are derived from the crystal structure of MaSp1(Ea) NT domain. The mutated amino acids and counterparts are boxed. The stars indicate the MaSp and Flag NT domains, which are mutated to NT^{*}_{MaSp} and NT^{*}_{FlSp}.

The sequences shown are the following: FlSp (Pt), *Parasteatoda tepidariorum* flagelliform spidroin variant 1, residues 14–146 (GenBank accession no. AWK58739); Flag (Nim), *Nephila inaurata madagascariensis* flagelliform silk protein, residues 1–154 (GenBank AAF36091, modified as in Ref. [1]); FlSp (NC), *Nephila clavipes* flagelliform spidroin variant 1, residues 25–154 (GenBank AWK58737); FlSp (Adi), *Araneus diadematus* flagelliform spidroin variant 2, residues 27–155 (GenBank AWK58735); FlSp (Aar), *Argiope argentata* flagelliform spidroin variant 1b, residues 27–156 (GenBank AWK58732); TuSp1(Sg), *Steatoda grossa* tubuliform spidroin 1 variant 1, residues 33–163 (GenBank AWK58644); TuSp1(Nc), *N. clavipes* tubuliform spidroin 1 variant 1, residues 24–152 (GenBank AWK58643); TuSp1(Na), *Nephila antipodiana* eggcase silk protein, residues 31–159 (GenBank ACI23395); TuSp1(Lh), *Latrodectus hesperus* tubuliform spidroin 1, residues 29–156 (GenBank ABD24296); TuSp1(Av), *Araneus ventricosus* tubuliform spidroin 1, residues 26–153 (GenBank ASO67373); TuSp1(Adi), *A. diadematus* tubuliform spidroin 1 variant 1, residues 26–132 (GenBank AWK58641); TuSp1(Aar), *A. argentata* tubuliform spidroin 1, residues 4–132 (GenBank ADM14332); TuSp1(Aap), *Agelenopsis aperta* tubuliform spidroin 1, residues 35–161 (GenBank ADM14330); CySp1(Ncl), *Nephila clavata* cylindrical silk protein 1, residues 31–159 (GenBank BAE54451); CySp1(Ab), *Argiope bruennichi* egg case silk protein 1, residues 26–154 (GenBank BAE86855); PySp(Nc), *N. clavipes* piriform spidroin protein PiSp, residues 23–144 (GenBank PRD25616); PySp(Aaq), *Argyroneta aquatica* piriform spidroin variant 2, residues 25–144 (GenBank AVH80558); PySp1(Sg), *S. grossa* pyriform spidroin 1 variant 1, residues 21–141 (GenBank AWK58661);

PySp1(Lh), *L. hesperus* pyriform spidroin 1 variant 1, residues 21–141 (GenBank AWK58659); PySp1(Aar), *A. argentata* pyriform spidroin 1, residues 22–143 (GenBank AQR58363); PySp1(Adi), *A. diadematus* pyriform spidroin 1 variant 1, residues 22–143 (GenBank AWK58658); AcSp(Sg), *S. grossa* aciniform spidroin 1 variant 1, residues 33–167 (GenBank AWK58693); AcSp(Pt), *P. tepidariorum* aciniform spidroin 1 variant 1, residues 33–171 (GenBank AWK58692); AcSp(Nc), *N. clavipes* aciniform spidroin 1 variant 1, residues 15–154 (GenBank AWK58691); AcSp(Av), *A. ventricosus* aciniform spidroin, residues 28–163 (GenBank AUH99620); AcSp(At), *Argiope trifasciata* aciniform spidroin 1 N-terminal region variant 5, residues 12–147 (GenBank AHK09780); AcSp(Adi), *A. diadematus* aciniform spidroin 1 variant 2, residues 28–163 (GenBank AWK58688); AcSp1(Lh), *L. hesperus* aciniform spidroin 1 variant 1, residues 33–167 (GenBank AWK58690); AcSp1(Lg), *Latrodectus geometricus* aciniform spidroin 1, residues 1–135 (GenBank AFX83566); AcSp1(Aau), *Argiope aurantia* aciniform spidroin 1 N-terminal region variant 6, residues 12–147 (GenBank AHK09775); AcSp1(Aar), *A. argentata* aciniform spidroin 1 N-terminal region variant 1, residues 12–147 (GenBank AHK09763); MiSp(Ud), *Uloborus diversus* minor ampullate spidroin, residues 14–141 (GenBank ADM14326); MiSp(Pt), *P. tepidariorum* minor ampullate spidroin, residues 22–150 (GenBank ARA91217); MiSp(Nc), *N. clavipes* minor ampullate spidroin protein MiSp-d, residues 22–148 (GenBank PRD18914); MiSp(Mg), *Metepeira grandiosa* minor ampullate spidroin, residues 16–143 (GenBank ADM14328); MiSp(Lh), *L. hesperus* minor ampullate spidroin variant 2, residues 5–133 (GenBank ARA91182); MiSp(Lg), *L. geometricus* minor ampullate spidroin, residues 5–133 (GenBank ARA91154); MiSp(Av), *A. ventricosus* minor ampullate spidroin, residues 22–149 (GenBank AFV31615); MiSp1(Sg), S. grossa minor ampullate spidroin 1 variant 1, residues 23–151 (GenBank AWK58682); MiSp1(Lh), *L. hesperus* minor ampullate spidroin 1 variant 1a, residues 23–151 (GenBank AWK58676); MiSp1(Adi), *A. diadematus* minor ampullate spidroin 1 variant 2, residues 22–149 (GenBank AWK58673); MaSp-like(Dc), *Diguetia canities* major ampullate spidroin-like protein, residues 19–147 (GenBank ADM14317); MaSp(Dc), *D. canities* major ampullate spidroin, residues 28–161 (GenBank ADM14315); MaSp(Aap), *A. aperta* major ampullate spidroin, residues 23–151 (GenBank ADM14324); MaSp2-like(Nim), *N. inaurata madagascariensis* major ampullate spidroin 2-like, residues 24–154 (GenBank AAZ15322); MaSp2(Pt), *P. tepidariorum* spidroin-2 isoform X9, residues 25–153 (GenBank XP_021002866); MaSp2(Nc), *N. clavipes* major ampullate spidroin 2 precursor, residues 24–154 (GenBank ACF19413); MaSp2(Lh), *L. hesperus* major ampullate spidroin 2, residues 28–154 (GenBank ABR68855); MaSp2(Lg), *L. geometricus* major ampullate spidroin 2, residues 1–124 (GenBank ABY67417); MaSp2(Ds), *Deinopis spinosa* major ampullate spidroin 2, residues 23–148 (GenBank ADM14319); MaSp2(At), *A. trifasciata* major ampullate spidroin 2, residues 25–155 (GenBank AAZ15371); MaSp2(Adi), *A. diadematus* major ampullate spidroin 2 variant 4, residues 22–152 (GenBank AWK58651); MaSp2(Ab), *A. bruennichi* major ampullate silk protein 2, residues 25–155 (GenBank AFN54363); MaSp2(Aar), *A. argentata* major ampullate spidroin 2 variant 2, residues 25–155 (GenBank AWK58646); MaSp1-like(Lg), *L. geometricus* major ampullate spidroin 1-like, residues 1–127 (GenBank AAZ15320, modified as in Ref. [1]); MaSp1B(Nc), *N. clavipes* major ampullate spidroin 1B precursor, residues 24–154 (GenBank ACF19412); MaSp1A(Nc), *N. clavipes* major ampullate spidroin 1A precursor, residues 24–154 (GenBank ACF19411); MaSp1(Sg), *S. grossa* major ampullate spidroin 1 variant 1, residues 25–151 (GenBank AWK58634); MaSp1(Pt), *P. tepidariorum* uncharacterized protein LOC107443970, residues 25–153 (GenBank XP_015913485); MaSp1(Lm), *Latrodectus mactans* major ampullate spidroin 1, residues 1–127 (GenBank ADO78764); MaSp1(Lh), *L. hesperus* major ampullate spidroin 1, residues 25–151 (GenBank ABR68856); MaSp1(Lg), *L. geometricus* major ampullate spidroin 1 variant 1 locus 2, residues 25–151 (GenBank ABY67428); MaSp1(Kh), *Kukulcania hibernalis* major ampullate spidroin 1, residues 25–156 (GenBank ADM14314); MaSp1(Adi), *A. diadematus* major ampullate spidroin 1 variant 1, residues 23–153 (GenBank AWK58624); MaSp1(Aar), *A. argentata* major ampullate spidroin 1 variant 1, residues 23–153 (GenBank AWK58623); MaSp1(Aaq), *A. aquatica* major ampullate spidroin variant 1, residues 30–160 (GenBank AVH80563); MaSp1(Ea), *Euprosthenops australis* Chain A, Self-Assembly Of Spider Silk Proteins Is Controlled By A Ph-Sensitive Relay, residues 5–135 (GenBank 3LR2_A).

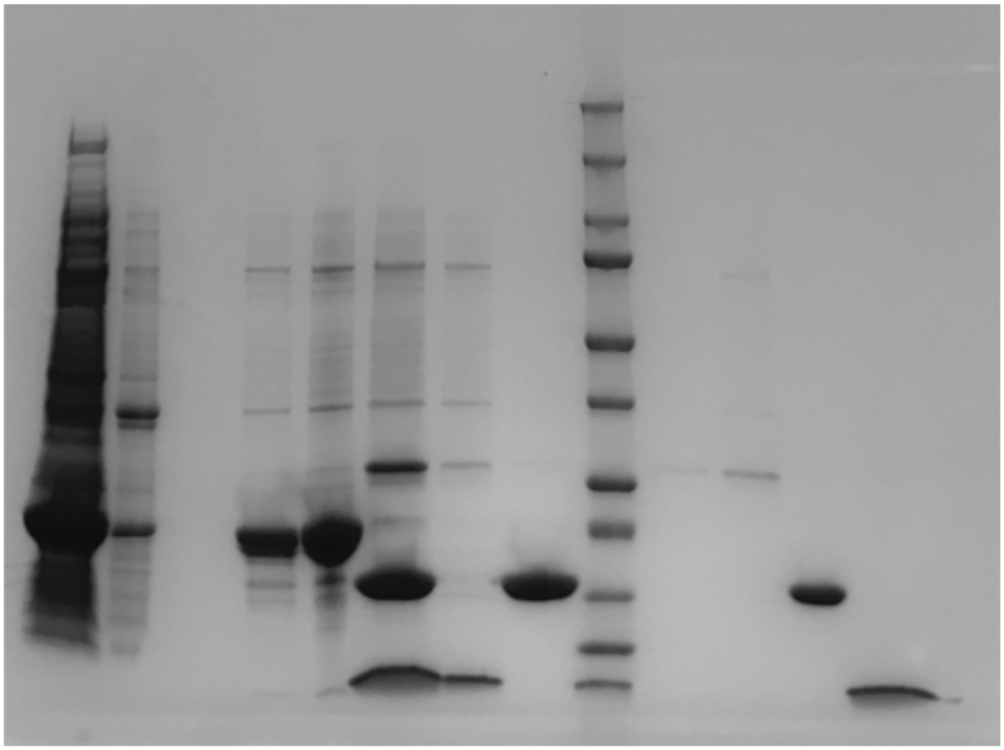

Supplementary Figure S2. Uncropped full-length gel image of Figure 2B with (1) lysate, (2) pellet, (3) IMAC elution with 300 mM Imidazole, (4) dialyzed fractions containing NT*$_{\mathrm{FlSp}}$TRS-Aβ_{42} from IMAC, (5) after TEV cleavage, (6) flow-through of reverse IMAC, (7) Elution of reverse IMAC, (8) void peak of SEC. The lanes (9-11) represent the fractions after SEC of (5), i.e. (9) TEV, (10) NT* and (11) Aβ. The protein ladder is marked by (L) and empty lane by (o).

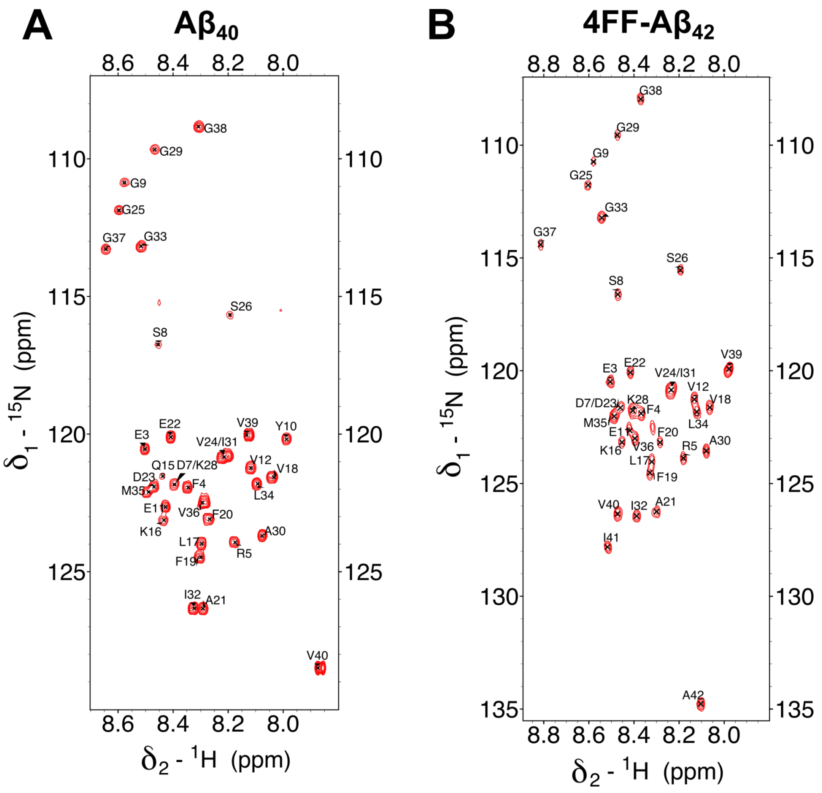

Supplementary Figure S3. ^{1}H-^{15}N HSQC spectrum of (A) 75 µM Aβ$_{40}$ in 16 mM sodium-phosphate buffer, pH 7.4, with 0.02% NaN_3 and 0.2mM EDTA, recorded at 8 °C and 500 MHz and (B) 15 µM 4FF-Aβ$_{42}$ in 20 mM sodium-phosphate buffer, pH 7.4, with 0.02% NaN_3 and 0.2 mM EDTA, recorded at 4 °C and 600 MHz.

SUPPLEMENTARY TABLES

Fitting parameter	Values for Aβ$_{42}$ aggregation kinetics
$\sqrt{k_n k_+}$	$4.08 \pm 0.17\ \mathrm{M^{-1}\ s^{-1}}$
$\sqrt{k_+ k_2}$	$5.34 \cdot 10^5 \pm 2\ \mathrm{M^{-3/2}\ s^{-1}}$
$\sqrt{K_M}$	3.71 ± 0.14 µM
$\boldsymbol{m(0)}$*	1.13±0.01; 1.37±0.01; 1.80±0.01; 2.11±0.01; 2.36±0.02; 3.30±0.03; 4.14±0.05; 4.97±0.08; 5.70±0.11; 6.70±0.16; 8.70±0.27

*$m(0)$ was constrained to ± 0.3 µM of the experimentally determined peptide concentration

Supplementary Table S1. Global fit parameter of Aβ$_{42}$ aggregation kinetics.
Errors are displayed as fitting errors.

REFERNCES

1 Rising, A., Hjalm, G., Engstrom, W. & Johansson, J. N-terminal nonrepetitive domain common to dragline, flagelliform, and cylindriform spider silk proteins. *Biomacromolecules* **7**, 3120-3124 (2006).
